# Effect of Corroded Surface Morphology on Ultra-Low Cycle Fatigue of Steel Bridge Piers

**DOI:** 10.3390/ma14030666

**Published:** 2021-02-01

**Authors:** Fangyuan Song, Tingting Zhang, Xu Xie

**Affiliations:** College of Civil Engineering and Architecture, Zhejiang University, Hangzhou 310058, China; cefysong@zju.edu.cn (F.S.); 3090103176@zju.edu.cn (T.Z.)

**Keywords:** seismic design of steel bridge, cyclic void growth model, steel corrosion, corrosion morphology, ultra-low cycle fatigue, finite element model

## Abstract

Corrosion is a common form of durability degradation of steel bridges. Corrosion morphology affects stress distribution under cyclic loads and causes strain concentrations in pits, thus affecting the mechanical properties of steel structures, including ultra-low cycle fatigue (ULCF). To precisely simulate corrosion morphology and investigate the ULCF failure mechanism of corroded steel piers, a sculpting method was applied to mesh units using three-dimensional surface morphology data of corroded steel specimens. Moreover, the ULCF crack-initiation life was numerically predicted using the finite element model based on the cyclic void growth model (CVGM). The cumulative equivalent plastic strain, cyclic void growth index, and critical void growth index of corroded steel piers with different corroded morphologies were compared. Results reveal that, regardless of whether the pier is corroded, fatigue cracks tend to initiate at the weld toe at corners when exposed to cyclic loads under an oblique direction at the pier top. Additionally, the ULCF crack-initiation life in a corroded pier is less than that in an uncorroded pier, and it is significantly affected by a reduction in the pier wall thickness. Corrosion pits affect the position of ULCF crack initiation in a steel pier and cracks tend to initiate at the bottom of pits with large depth-to-diameter ratios. In the case of minor corrosion, the corrosion morphology affects the seismic performance of piers to a small extent.

## 1. Introduction

Ultra-low cycle fatigue (ULCF) damage is the main failure form of steel pier structures under a strong earthquake due to large seismic plastic cyclic strain [1,2,3]. Therefore, ULCF in steel piers needs to be well understood to optimize the seismic design of steel-pier structures. Since the 1995 Great Hanshin–Awaji Earthquake in Japan, numerous studies have been conducted on the failure mechanism and methods to calculate the ULCF life in steel structures. In one of these works, Ge et al. [4,5,6] proposed an evaluation method for predicting the ductile crack initiation of steel structures against ultra-low cycle fatigue by using the strain concentration factor, Miner’s fatigue damage accumulation theory, and the Coffin–Manson equation.

In other studies, different models have been proposed to numerically calculate the ULCF in steel structures. Kanvinde and Deierlein [7,8] considered void growth and coalescence theories to propose the cyclic void growth model (CVGM) and degraded significant plastic strain model for simulating ductile-fracture initiation due to large-amplitude cyclic strains in structural steels. Wang et al. and Zhou et al. [9,10,11] used the CVGM to predict ultra-low cycle fatigue fracture of the beam-to-column connections under inelastic cyclic loadings. Fell et al. [12] verified the accuracy of CVGM calculations by comparing them with experimental results. Bonora [13] used the continuum damage model (CDM) to analyze the growth of micro-voids resulting from nonlinear damage accumulation upon plastic deformation of the material constitutive. CDM is a nonlinear plasticity damage model for predicting fatigue life and is based on the equivalent strain principle. Tong et al. [14] used the CDM to predict the ULCF crack-initiation life of Q345qC steel and weld joints. Tian et al. [15,16] simulated ULCF fractures in steel specimens and verified the accuracy of CDM.

Until now, significant research has focused on the failure mechanism of ULCF fracture and subsequent crack initiation in steel [17,18,19,20]. The consensus is that brittle fracture after ductile crack initiation occurs under large-strain cyclic loading. For unstiffened cantilever steel columns with large cyclic deformation, Tateishi et al. [21,22] monitored the process of crack initiation, propagation, and final failure during ULCF fracture tests and proposed an approach based on the local strain to estimate the ULCF crack-initiation life. In addition, they investigated the extremely low cycle fatigue life of such columns. Chi and Gao [23] proposed an empirical formula to calculate the initiation life of ULCF cracks in thick-walled steel piers by applying the Ge model [24], a plastic strain-based evaluation method for predicting the ductile crack initiation in steel structures. Xie et al. [25] also proposed a numerical approach based on the deformation history to evaluate ULCF damage in steel structures. These studies form a foundation for seismically designing sound steel piers that consider the ULCF strength.

However, the mechanical performance of steel structures is significantly degraded by corrosion. Thus, the evaluation of the seismic performance of in-service steel piers must account for corrosion. In addition to reducing the effective cross-sectional area, corrosion concentrates strain at pits, which shortens the crack-initiation life in ULCF, thereby reducing the seismic performance of the structure. Previous studies have examined, both experimentally and theoretically, ULCF failure in corroded steel bars. For example, Apostolopoulos [26] tested the mechanical performance of corroded reinforcing steel bars under large strain cyclic load and found that the corroded steel bars gradually lose both their load-bearing ability and available energy. By measuring the ULCF life of corroded steel plates in a laboratory, the authors [27] found that the strain concentration at corrosion pits promotes the initiation of ULCF cracks and shortens the fatigue life of steel plates. However, this is one of the few studies on the ULCF strength of corroded steel plates. To date, the effect of corrosion on steel bridge piers’ ULCF properties has not been comprehensively understood through either experiment or numerical study, the corresponding research for crack initiation life and position is limited; hence, another study on the seismic performance of steel structures is warranted.

This paper aims to study ULCF in corroded steel piers, thus we raised up a sculpting method to mesh units using three-dimensional surface morphology data of corroded steel specimens to precisely simulate the corrosion morphology of different corroded surfaces and numerically predicted the ULCF crack-initiation life using the finite element model (FEM) based on CVGM. This approach improves the use of artificial, regular geometric shaped pits on finite element models in the existing studies, and allows us to analyze how pit morphology affects crack initiation and provides a reference for evaluating the seismic performance of corroded steel piers in active service.

## 2. Structural Parameters and ULCF Failure Simulation

### 2.1. Structural Parameters

**Figure 1:**
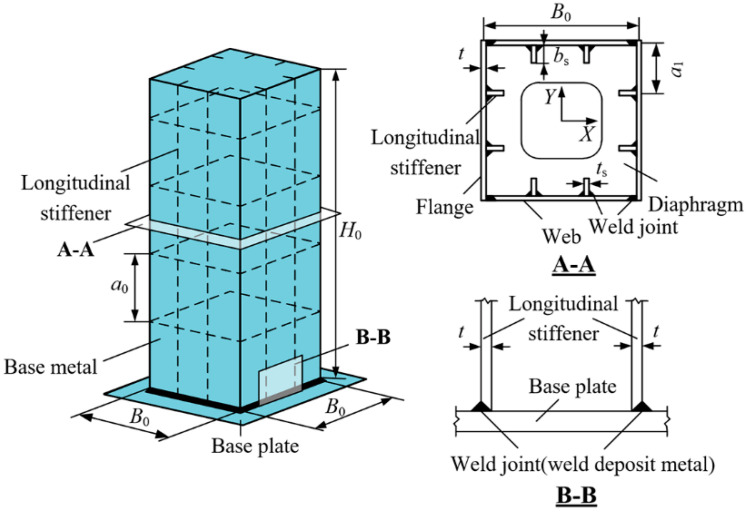
Schematic of a single-column steel bridge pier.

To investigate how corrosion morphology affects the ULCF resistance of corroded steel piers, we use a single-column steel bridge pier as research object (see Figure 1). Table 1 lists the geometric dimensions of the steel pier. The pier is made of Q345qC steel, which is widely used in bridge structures, and its constitutive relation is based on the Lemaitre–Chaboche [28] hybrid hardening model. The size of the yield surface σ^0^ can be expressed with a simple exponential law as

(1) $$\sigma^{0}=\sigma {|}_{0}+Q_{\infty }(1-e^{-b\varepsilon_{p}})$$

where σ|_0_ is the yield surface size at zero plastic strain, *Q*_∞_ is the maximum change value of yield surface, and *b* is the changing rate of the yield surface as plastic strain develops. *Q*_∞_ and *b* are additional material parameters that can be calibrated from cyclic test data. 

The overall backstress *α* is composed of multiple backstress components *α_k_*, where the evolution of the backstress components *α_k_* of the model is defined as

(2) $$\alpha_{k}=\frac{C_{kin,k}}{\gamma_{k}}(1-e^{-\gamma_{k}\varepsilon_{p}})+\alpha_{k,1}e^{-\gamma_{k}\varepsilon_{p}}$$

where *C_kin,k_* is the initial values of the kinematic hardening modules, and *γ*_k_ is the reduction rate of *C_kin,k_* with increasing plastic strain. Overall backstress *α* is calculated from the relation

(3) $$\alpha =\sum_{k=1}^{N}\alpha_{k}$$

The relevant cyclic hardening parameters of Q345qC steel are given in Table 2 [29,30], where *k* = 3, and the material parameters were calibrated by Wang [29] and Liao [30] from cyclic test data. Given that ULCF crack-initiation life strongly affects the welding properties, the materials of the steel pier are divided into two types of metals: A base metal and weld-deposit metal. 

### 2.2. Corrosion Morphology Simulated Finite Element Model

Coating degradation, small gaps introduced at joints, etc., can induce an electrochemical reaction between the steel and surrounding medium to corrode the structure. The corrosion of the steel plate can be divided into uniform and pitting corrosion. The former usually decreases the thickness of the steel plate, and the latter often pits the surface of the steel plate.

**Figure 2:**
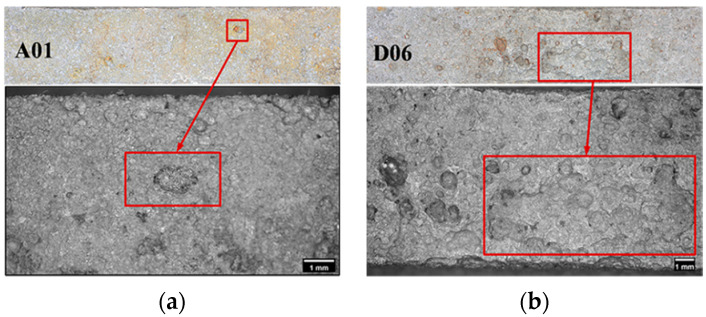
Surface morphology of corroded specimens: (**a**) Corrosion time: 60 days; (**b**) Corrosion time: 240 days [31].

Experiments have been performed to show that corrosion pits serve as the initiation points for fatigue cracks and shorten the ULCF crack-initiation life [27]. Therefore, the surface morphology of the corroded steel (e.g., the size and distribution of pits) significantly affects ULCF crack initiation and propagation. Figure 2 compares the surface morphologies of specimens corroded under different times. A shorter corrosion time results in fewer pits on the surface of the specimen; hence, the pits consequently being less dense on the surface. As corrosion time increases, the pits become more densely packed on the surface; some pit communities are formed in which pits overlap. While the corroded area increases over time, the aspect ratio of the pits on the steel surface remains essentially the same [31].

To investigate how the corrosion morphology affects the ULCF performance of square steel piers, a precise simulation of the pitting morphology of the steel surface is warranted.

The FEM based on the CVGM was established to numerically calculate the ULCF crack-initiation life. A submodel was implemented in ABAQUS to improve the calculation accuracy of ULCF fractures while limiting the number of elements in FEM.

**Figure 3:**
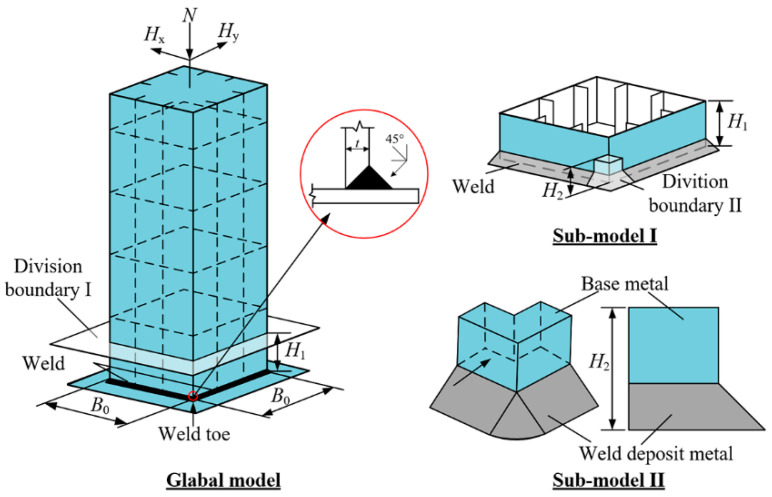
Finite element model separated into submodels.

**Figure 4:**
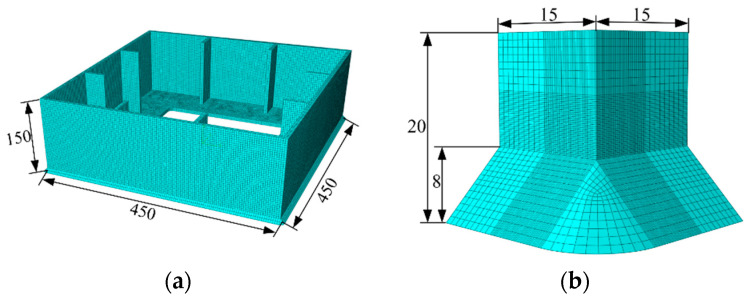
Mesh and submodels: (**a**) Sub-model I (unit: mm); (**b**) sub-model II (unit: mm).

As shown in Figure 3, two refined pier submodels were established to describe the stress distribution in the corner of a square and thin-walled pier as the ULCF crack initiates in the weld toe under seismic loading in two horizontal directions. The FEM comprises a global model, submodel I, and submodel II. Submodel I is a part of the global model and extends from the base of the pillar up to a height of 0.15 m (*H*_1_ = 0.15 m, see Figure 3). Submodel II is a part of submodel I and covers the weld toe in the corner, which is predicted to be a critical position under the ULCF load. In particular, submodel II extends from the base up to a height of 0.02 m (*H*_2_ = 0.02 m, see Figure 3). The global model uses four-node iso-parametric shell elements (S4R) and beam elements (B31). Along the thickness direction, the shell elements have five integration points and the element dimension is approximately 30 mm. Submodel I uses eight-node hexahedral elements (C3D8R), approximately 3.5 mm in size, and submodel II uses solid C3D8R elements as well. Table 3 lists the meshing information and Figure 4 shows the mesh units and submodels. Note that the minimum element size of submodel II is approximately 0.2 mm based on the characteristic length parameter of the steel material. A mean (or expected) characteristic length of 0.2 mm was determined for mild A579 Grade 50 steel as the average size of the plateaus and valleys based on 15 measurements from micrographs [32]. Furthermore, Liao et al. [33] calibrated the characteristic length of Q345 steel in China through tensile and cyclic load tests, and proposed the mean value of the parameter is around 0.2 mm. while another Liao [30] found the mean value of characteristic length is from 0.21 mm to 0.29 mm. Therefore, the minimum size of submodel II defined by 0.2 mm is sufficient in consideration of characteristic length. 

**Figure 5:**
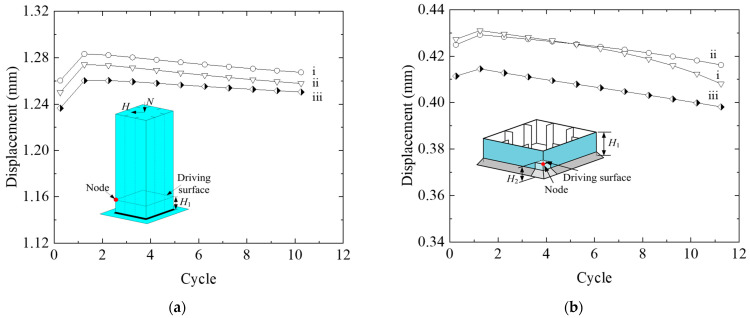
Sensitivity of the mesh size on the numerical results: (**a**) Global model; (**b**) submodel I.

In order to examine the mesh dependency of global model and submodel I, numerical analysis on model convergence and influence of the mesh size on the results was conducted. Table 4 lists the calculated models with different meshing sizes. As described by submodeling technology, the boundaries of submodel I was driven by the time-dependent displacements of nodes on driving surface that were saved during the previous analysis of the global model, and so was submodel II. Therefore, Figure 5a,b compared the displacement results at nodes on the driving surface of global model and submodel I, respectively.

As shown in Figure 5a, the peak deformation of the node in red on driving surface at each load cycle gradually increases as element size decreases. Despite this, the average peak deformation of global model ii is about 0.7% and 1.5% deviation from that of global models i and iii, respectively, indicating a sufficient element size of 30 mm (global model ii) in consideration of convergency and calculation efficiency. Similar conclusion can be drawn in Figure 5b, the peak deformation curves of submodel I i and submodel I ii tend to coincide with each other, and this illustrates the high computational convergence of the FE model with element size of 3.5 mm.

**Figure 6:**
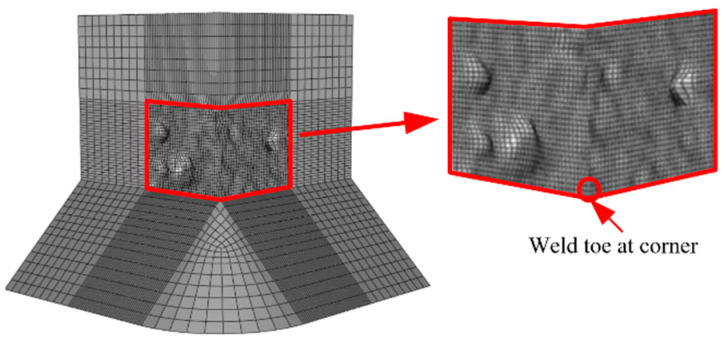
Corrosion morphology included in a sample.

To precisely simulate the corrosion morphology, we applied the three-dimensional surface morphology data obtained from corroded steel specimens to submodel II of FEM using the sculpting function of HyperMesh. Figure 6 shows a sample of submodel II with the corrosion morphology included; the red frame shows the morphology of the pits. All subsequent simulations were performed at the corner.

### 2.3. CVGM to Evaluate ULCF Strength

A ULCF fracture is characterized by the initiation of a ductile crack. ULCF strength is evaluated in two main ways: The first is the empirical method based on the Coffin–Mansion equation, and the second is a semi-empirical and semi-theoretical method based on a microscopic damage mechanism and includes the CVGM and CDM. Because the Coffin–Manson equation does not reflect how triaxial stress affects the ULCF, we use the CVGM method to calculate the steel pier’s ULCF crack-initiation life. The CVGM defines the void growth index under cyclic loading, *VGI*_cyclic_, and the critical void growth index, *VGI*_critical_, as

(4) $$VGI_{\mathrm{cyclic}}=\sum_{\mathrm{tensile}}\int_{\varepsilon_{1}}^{\varepsilon_{2}}\exp\left(\left|1.5T\right|\right)d\varepsilon_{p}^{t}-\sum_{\mathrm{compressive}}\int_{\varepsilon_{1}}^{\varepsilon_{2}}\exp\left(\left|1.5T\right|\right)d\varepsilon_{p}^{c}$$

(5) $$VGI_{\mathrm{critical}}=\eta \exp\left(-\lambda \varepsilon_{p}^{acc}\right)$$

A ULCF fracture occurs when

(6) $$VGI_{\mathrm{cyclic}}=VGI_{\mathrm{critical}}$$

In Equations (4)–(6), *T* is the stress triaxiality, $\varepsilon_{p}^{acc}$ is the cumulative equivalent plastic strain at the beginning of each tension cycle, $d\varepsilon_{p}^{t}$ and $d\varepsilon_{p}^{c}$ are the increment of equivalent plastic strain at each tension and compression cycle, respectively, *λ* is the damage degradation parameter of materials, and *η* is the fracture parameter under monotone loading. This paper adopts the CVGM fracture criterion calibrated by Li [34] to calculate the ULCF crack-initiation life of corroded steel piers at the corner of the weld toe. The criterion divides different applications of the damage degradation parameter by the average stress triaxiality. When *T* > 0.70, *λ* = −0.08, and when 0.33 < *T* ≤ 0.70, *λ* = −0.18. The average stress triaxiality *T_m_* is

(7) $$T_{m}=\varepsilon_{F}{}^{-1}\int T\left(\varepsilon_{p}\right)d\varepsilon_{p}$$

where *ε*_F_ is the fracture strain of notched specimens at the instant of crack initiation, and *T*(*ε_p_*) is the loading history of stress triaxiality obtained by FEA.

The ULCF strength is a mechanical property that is based on the characteristic length. To predict ULCF crack initiation, the FEM requires a probable element dimension of approximately 0.2 mm in the crack region. However, using elements smaller than 0.2 mm for the global model creates too many elements and renders the computation unwieldy. Hence, we applied a submodel to establish refined FEMs for simulating the corroded surface morphology.

## 3. Results and Discussion for ULCF Strength

### 3.1. Reliability of Numerical Simulation

The reliability of the FEM was verified by comparing the numerical calculations with experimental results that conducted by Zhuge [35].

**Figure 7:**
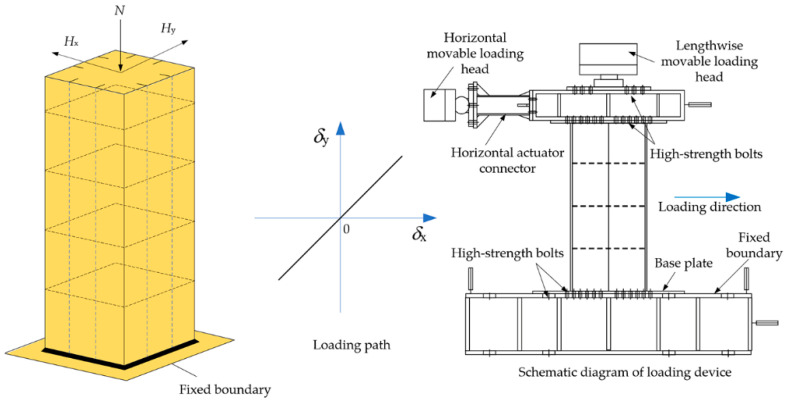
Loading path.

As laboratorial experiment designed by Zhuge, a constant axial force *N* and two cyclic bidirectional horizontal enforced displacements were applied to the top of the pier to produce the loading pattern for simulating the ULCF fracture of the steel pier (Figure 7). The bottom of the pier was fixed to the ground, and the axial compression ratio was 0.2. The cyclic horizontal load included bidirectional horizontal thrusts *H*_x_ and *H*_y_ (enforced displacement) that compose the total load in the direction along the center to corner of the plane. Laboratory tests and numerical calculations [35] have demonstrated that the bearing capacity of the steel pier structure is lowest along the oblique direction among possible loading paths, and the ULCF crack initiates at the corner of the weld toe. Therefore, the same loading path was applied in this study for computation and comparison.

**Figure 8:**
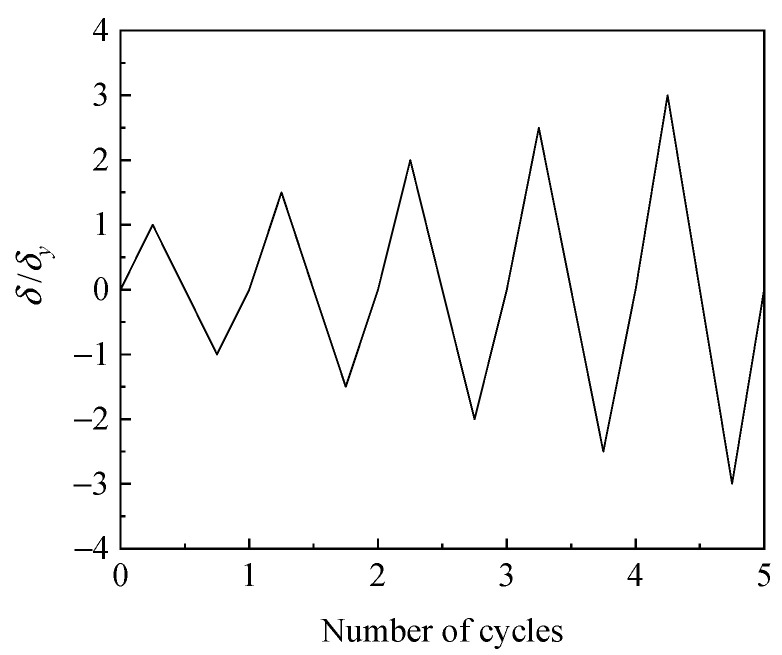
Loading pattern I.

The loading pattern in the experiment is shown in Figure 8. The axial pressure and bending moment produce definite yield displacement *δ_y_* at the weld toe. As the load increases, the yield displacement is incremented by 0.5*δ_y_* in each cycle, where *δ_y_* is obtained by applying mechanical theory to the frame structure [35]. The horizontal yield thrust *H*_yield_ can be obtained by calculating the minimum of

(8) $$\left\{ \begin{array}{l} H_{y\mathrm{ield}}=\frac{M_{y}}{h}(1-\frac{N}{N_{y}})\\ H_{y\mathrm{ield}}=\frac{M_{y}}{0.85h}(1-\frac{N}{N_{E}})(1-\frac{N}{N_{u}}) \end{array}\right.$$

(9) $$\delta_{y}=\frac{H_{yield}h^{3}}{3EI}+\frac{H_{yield}h}{\kappa GA}$$

where *M_y_* is the yield bending moment of the steel pier, *N_E_* is the Euler’s buckling load of a cantilever column, *N_u_* is the ultimate strength of steel piers, *N_y_* is the yield axial force, *G* is the shear modulus, *A* is the cross-sectional area, *κ* is the shear unevenness coefficient of cross section, which is taken as 5/6 herein, and *h* is the height of the cantilever column, *I* is the moment of inertia.

**Figure 9:**
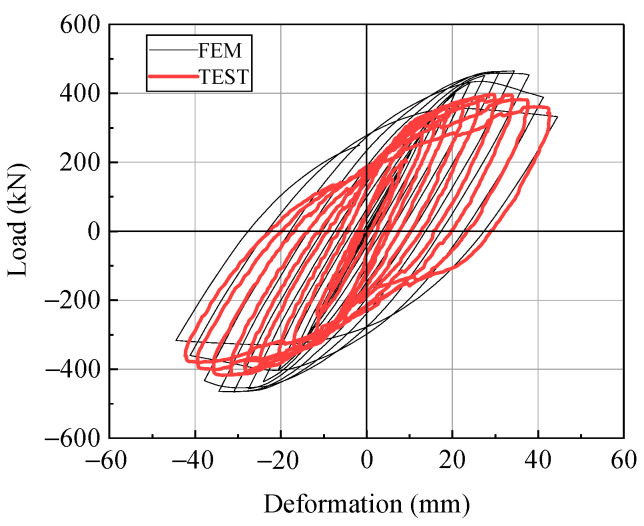
Load-deformation of specimens obtained by experiment and finite element model.

In order to verify the reliability of FE model that raised in Section 2.2, Figure 9 compares the force–displacement results of the experiments [35] and FEM simulation in this study. The two curves overlap well. The precision of the cyclic hardening model was slightly less than that of the two-surface constitutive model, which led to increased structural stiffness after yielding. Thus, the FEM results were less precise than the experimental results.

### 3.2. Influence of Corroded Surface Morphology

**Figure 10:**
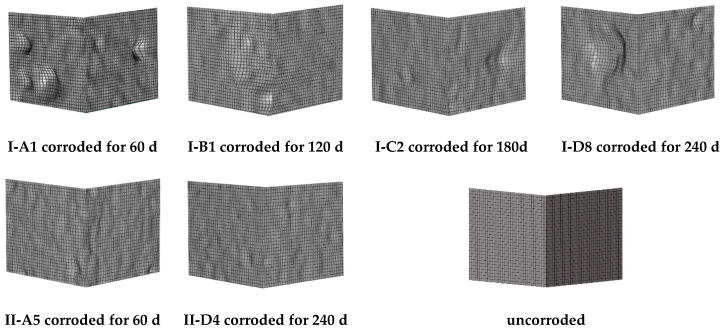
Simulated corners of the steel bridge pier showing damage due to different corrosion times.

The three-dimensional corrosion morphology obtained by scanning the steel specimens (using a salt spray test) is simulated by FEM [31]. Groups labeled A–D indicate models of specimens corroded for 60, 120, 180, and 240 days and with average mass losses of 7.24%, 9.61%, 9.79%, and 10.59%, respectively. The specimens are further divided into groups I and II, with group I specimens having significant corrosion pits, which is characteristic of typical pitting corrosion, while group II specimens have an even distribution of minor pits, which is characteristic of uniform corrosion. Figure 10 shows the simulated surface morphology at the foot of the pier for specimens with different corrosion times.

**Figure 11:**
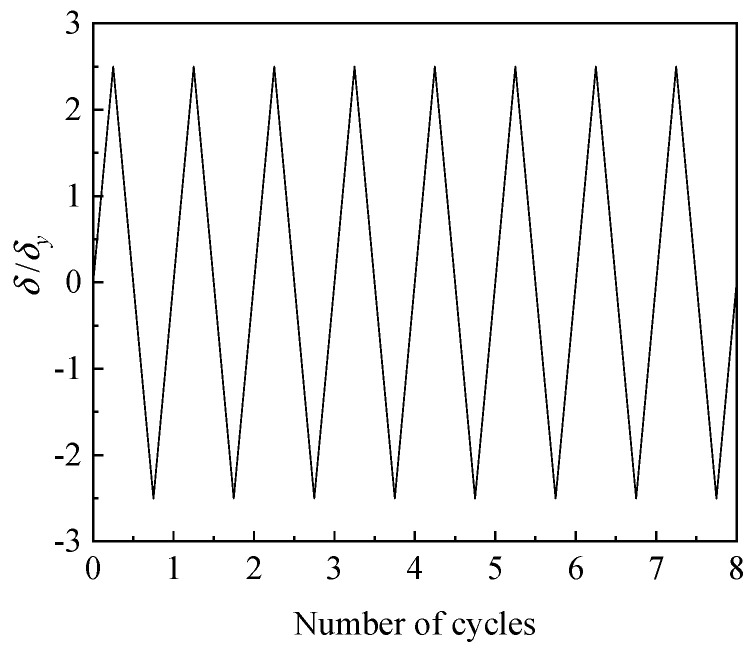
Loading pattern II.

To promote a ULCF fracture in the numerical simulation, the loading path and axial compression ratio were the same as that described in Section 3.1 (see Figure 7). The loading pattern is shown in Figure 11.

**Figure 12:**
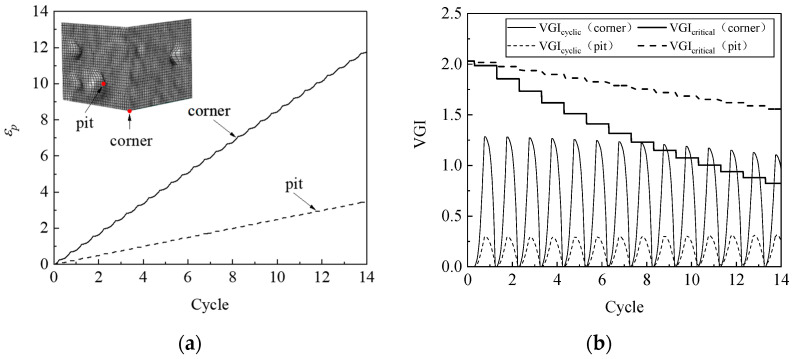
*ε_p_*, *VGI*_cyclic_, and *VGI*_critical_ at the corner and corrosion pit of pier A1: (**a**) *ε_p_*; (**b**) *VGI*_cyclic_ and *VGI*_critical._

The simulation results indicate that a ULCF crack initiates at the weld toe at the corner of the pier for various corrosion morphologies, which is consistent with the crack-initiation position for the uncorroded pier. As an example, Figure 12 compares the accumulated equivalent plastic strain *ε_p_*, the cyclic void growth index *VGI*_cyclic_, and the critical void growth index *VGI*_critical_ as a function of strain cycle for corroded steel pier A1 at the corner weld toe versus that at the bottom of a pit. The growth rate of *ε_p_* and the peaks of *VGI*_cyclic_ at the weld toe significantly exceed those at the bottom of the pit. However, *VGI*_critical_ at the weld toe decreases more rapidly, so the crack initiates at cycle 8.7, which is when *VGI*_critical_ reaches the critical point at the weld toe.

**Figure 13:**
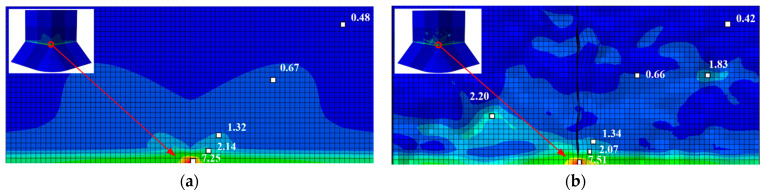
Distribution of equivalent plastic strain *ε_p_* at the weld toe corner of the pier: (**a**) *ε_p_* of uncorroded pier; (**b**) *ε_p_* of corroded pier A1 when cracked.

Figure 13 compares the *ε_p_* distribution around the corner at the foot of an uncorroded pier with the same for corroded pier A1. Independent of the state of corrosion, the strain concentrates near the weld toe at the pier corner. The strain *ε_p_* decays rapidly with horizontal and vertical distance from the corner position, and the strain is less concentrated at the pit, reducing the likelihood of ULCF crack initiation at the corner.

Table 5 shows the ULCF crack-initiation life obtained by simulation of piers with different corrosion times. The ULCF crack-initiation life for the uncorroded pier is 8.7, which remains unchanged for corroded piers A1 and B1. However, the ULCF crack-initiation life of piers C2 and D8 decreases remarkably by about 23% and 46%, respectively. Therefore, the ULCF crack-initiation life of corroded piers declines only slightly when corrosion degradation is not noticeable, so the seismic design of structures need not consider corrosion in this case. In addition, the simulation indicates that the ULCF crack-initiation life of the steel pier is not directly correlated with corrosion mass loss, which is consistent with the results of numerical simulations of steel specimens [27]. The ULCF crack-initiation life of piers A5 and D4, which have a relatively even surface-corrosion morphology, are 3.7 and 7.7 cycles, respectively. Despite the pits not being distributed with a high degree of homogeneity on the surface, the corrosion affects the ULCF life. Thus, both uniform corrosion and pitting corrosion degrade the ULCF performance of the steel pier.

### 3.3. Effect of Uniform Corrosion on ULCF Life

**Figure 14:**
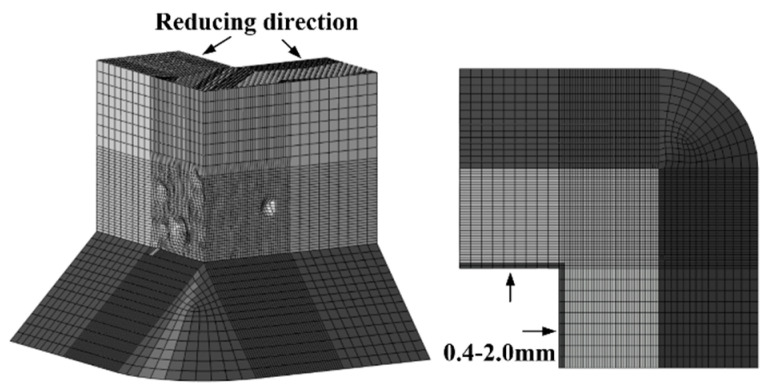
Schematic showing the reduction in the wall thickness of steel bridge pier A1.

To investigate how uniform corrosion affects the ULCF crack-initiation life of a thin-walled square steel pier, we simulate piers with walls of different thicknesses. For pier A1, we reduce the wall thickness, thus lessening the cross-sectional area to simulate uniform corrosion, as shown in Figure 14. Four specimens are considered with wall thickness reduced by 0, 0.4, 1.2, and 2.0 mm (corresponding to a wall thinning of 0%, 5%, 15%, and 25%).

**Figure 15:**
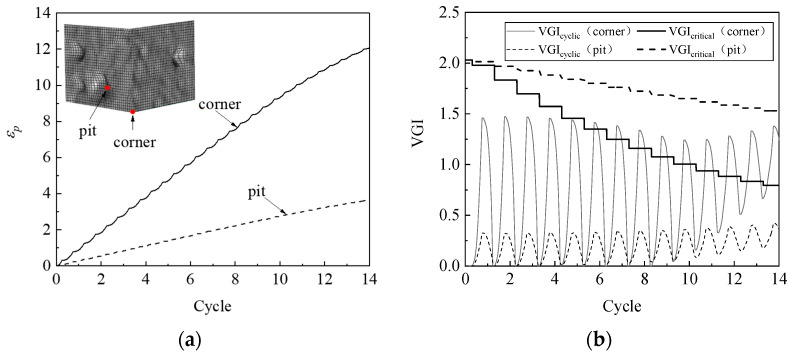
*ε_p_*, *VGI*_cyclic_, and *VGI*_critical_ for the corner and corrosion pit of pier A1 when the wall was thinned by 0.4 mm: (**a**) *ε_p_*; (**b**) *VGI*_cyclic_ and *VGI*_critical._

ULCF cracks still occur at the corner in the various square, thin-walled steel piers. For example, Figure 15 compares *ε_p_*, *VGI*_cyclic_, and *VGI*_critical_ vs. cycle number at the corner with that in the pit for pier A1 with its walls uniformly thinned by 0.4 mm. The accumulated equivalent plastic strain *ε_p_* is greater at the corner than in the pit, similar to the peak value of *VGI*_cyclic_. However, *VGI*_critical_ decreases more rapidly as a function of cycle number at the corner than in the pit, contributing to earlier crack initiation at the corner.

Table 6 lists the ULCF crack-initiation life obtained by simulating steel piers with uniform corrosion. The ULCF crack-initiation life of corroded piers decreases significantly with decreasing wall thickness; for wall thickness reductions exceeding 15%, the ULCF crack-initiation life is about 1.8 cycles. These results suggested that ULCF degrades material strength mainly by reducing the cross-sectional area, thereby leading to earlier ULCF fracture in piers.

### 3.4. Influence of Pitting Size and Location

**Figure 16:**
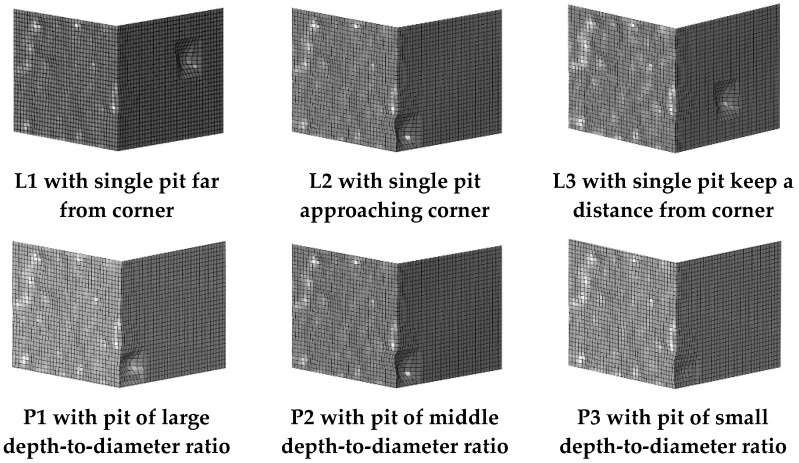
Corner at weld toe with single corrosion pit.

To further investigate how pitting affects ULCF crack-initiation life for square steel piers, we simulate the pier surface with corrosion pits whose size and position are based on the experimentally observed pit morphology of steel specimens. Furthermore, the competitive mechanism between the corner and pit of ULCF crack initiation is studied. Given that the corner at the weld toe is the most fragile point of the pier structure, we position a pit at the corner. As shown in Figure 16, identical pits mar the surface of models L1–L3 (albeit at different positions). Another pit with a varying depth-to-diameter ratio is placed on the surface of models P1–P3 at the same position as for model L2 (corresponding to depth-to-diameter ratios of 0.41, 0.36, and 0.17). Note that the pit shape and size were obtained by scanning an actual corroded specimen.

Table 7 lists the results of a simulation of the ULCF properties (including crack-initiation position and ULCF crack-initiation life) for a steel pier with a single pit at various positions on the surface (models L1−L3) and with a single pit at the corner with varying depth-to-diameter ratio (models P1–P3). All cracks initiate at the corner except for model P1, where a ULCF fracture initiates in the pit. These results indicate that only when a critical pit is close to the corner and has a sufficiently large depth-to-diameter ratio does a crack initiate in the pit due to an ULCF fracture.

**Figure 17:**
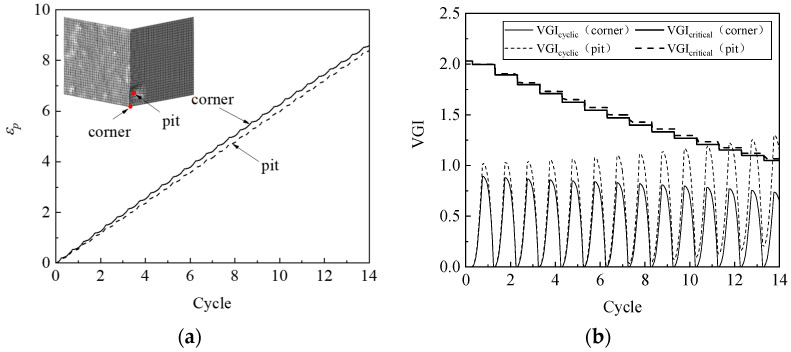
Comparison of *ε_p_*, *VGI*_cyclic_, and *VGI*_critical_ at the corner and corrosion pit of pier P1: (**a**) *ε_p_*; (**b**) *VGI*_cyclic_ and *VGI*_critical._

To uncover the mechanism that determines whether a crack initiates at the corner or in a pit, Figure 17 compares *ε_p_*, *VGI*_cyclic_, and *VGI*_critical_ for a steel pier under an ULCF load. While *ε_p_* has similar growth rates at the bottom of the pit and corner, the peaks of *VGI*_cyclic_ are remarkably greater in the pit. Conversely, *VGI*_critical_ is almost the same at the corner and in the pit. As a result, the crack initiates earlier in the pit, leading to an ULCF fracture at cycle 6.7.

**Figure 18:**
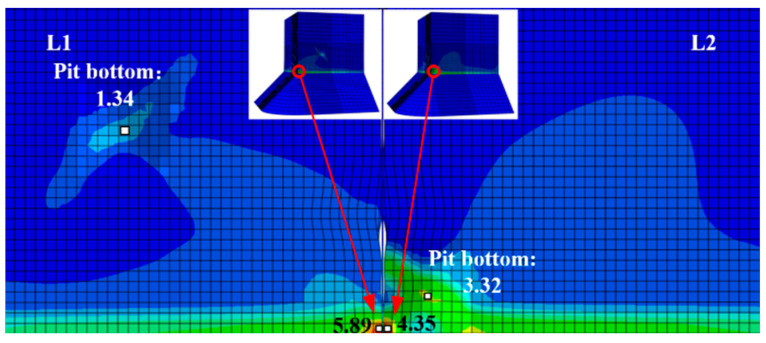
Distribution of equivalent plastic strain *ε_p_* at the corner of corroded piers of models L1 and L2.

The ULCF life of a corroded pier is shorter than that of an uncorroded pier (Table 7). For example, the ULCF crack-initiation life for pier L2, with a critical pit near the corner, is significantly less (5.7 cycles) than for the same pier with the pit far from the corner. This result suggests that the shortened ULCF crack-initiation life of corroded piers is related to pits being near the corner where the strain concentrates strongly under cyclic loads. Figure 18 shows the distribution of *ε_p_* for piers L1 and L2. When the pit is far from the corner (model L1), *ε_p_* is much smaller near the pit, so less strain is concentrated at the pit. In the opposite scenario (model L2), the pit concentrates significant strain at the corner.

**Figure 19:**
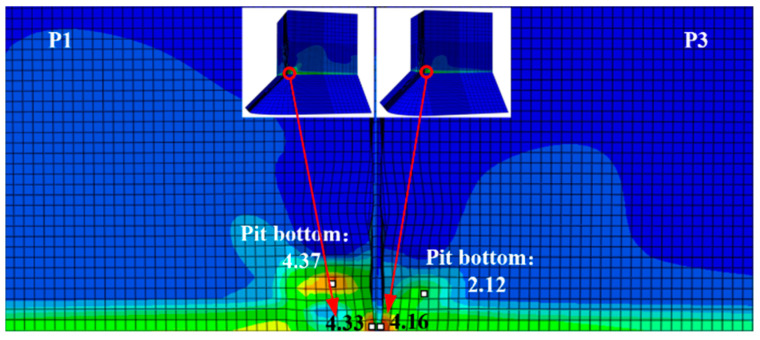
Distribution of equivalent plastic strain *ε_p_* at the corner of corroded piers of models P1 and P3.

Comparing the ULCF crack-initiation life of piers with pits of different depth-to-diameter ratios shows that the ULCF crack-initiation life increases with the ratio. The corner at the weld toe is the most fragile point of the pier structure where a strongly concentrated strain and stress occur under a cyclic load (see Figure 19). Thus, these results suggest that only when the pit (i) has a sufficient depth-to-diameter ratio and (ii) is close to the corner does it concentrate sufficient strain at the corner, relieve the corner’s stress, and final happen ULCF crack initiation, by which the pier structure raises its seismic load-bearing capacity. Thus, piers with a larger depth-to-diameter ratio have a longer ULCF crack-initiation life than those with small depth-to-diameter ratios.

## 4. Conclusions

This paper uses a FEM (with submodels) based on the measured surface morphology of corroded steel specimens and cyclic void growth theory to analyze how surface morphology affects the ULCF characteristics of a corroded steel bridge pier.

The results indicate that the pier corner and welded joint in a thin-walled square steel pier are prone to ULCF crack initiation because plastic strain is concentrated on these positions, even when the pier is corroded.

In addition, the ULCF crack-initiation life of thin-walled square steel piers is reduced by both pitting corrosion and uniform corrosion. The ULCF crack-initiation life decreases gradually when the corrosion rate is low. However, the UCLF crack-initiation life dramatically decreases as the pier wall thickness decreases. When the pier wall is thinned more than 15% by uniform corrosion, the ULCF crack-initiation life of the steel pier drastically decreases to 21%.

Finally, corrosion pits affect the position of ULCF crack initiation in a steel pier. Cracks will initiate at the bottom of pits with large depth-to-diameter ratios and that are close to the corner of the pier. The closer a pit is to the corner, the more it reduces the ULCF life of the structure. However, pits with greater depth-to-diameter ratios somewhat relieve the concentration of strain at the bottom corner of the pier, thereby reducing the shortening effect on the ULCF crack-initiation life of the structure compared to pits with small ratios.

Given these results, sharp corners should be avoided in the design of square steel bridge piers. In addition, the ULCF crack-initiation life and seismic performance of the structure can be improved by increasing the thickness of the pier wall at the bottom of the pier near the weld joint. Moreover, if corrosion is slight, corrosion morphology can be ignored when evaluating the seismic performance of the structure. However, the plate thickness should be reduced by 15% to verify the calculated seismic performance of a structure.

## Figures and Tables

**Table 1:** Geometric dimensions of steel bridge pier.

*B*_0_ (m)	*H*_0_ (m)	*t* (mm)	*t_s_* (mm)	*b_s_* (mm)	*a*_0_ (m)	*a*_1_ (m)
0.45	2.0	8.0	8.0	49	0.45	0.15

**Table 2:** Cyclic hardening parameters of Q345qC steel [29,30].

Material	*σ*|_0_ (MPa)	*Q*_∞_ (MPa)	*b*	C_kin,1_ (MPa)	C_kin,2_ (MPa)	C_kin,3_ (MPa)	γ_1_	γ_2_	γ_3_
Base metal	391.2	21.0	10	1800.0	1800.0	1800.0	245.0	155.0	50.0
Weld-deposit metal	428.45	17.4	0.4	12,752.3	1111.2	630.5	160.0	160.0	26.0

**Table 3:** Summary of meshing information.

	Global Model	Submodel I	Submodel II
Element type	S4R & B31	C3D8R	C3D8R
Element size	30 mm	3.5 mm	0.2 mm
No. of elements	6026	49,412	108,921

**Table 4:** Griding partition details of global model and submodel I.

No.	Element Size	No. of Elements	No.	Element Size	No. of Elements
Global model i	10 mm	49,016	Submodel I i	2.5 mm	161,632
Global model ii	30 mm	6026	Submodel I ii	3.5 mm	49.412
Global model iii	50 mm	2096	Submodel I iii	7.0 mm	28,302

**Table 5:** Results of simulation of ultra-low cycle fatigue (ULCF) crack-initiation life of corroded steel bridge piers.

	Uncorroded	A1	B1	C2	D8	A5	D4
ULCF life	8.7	8.7	8.7	6.7	4.7	3.7	7.7
*T*m	0.76	0.73	0.69	0.61	0.69	0.92	0.78

**Table 6:** Results of simulation of ULCF crack-initiation life of steel piers with uniform corrosion.

	Uncorroded	A1, 0.4 mm	A1, 1.2 mm	A1, 2.0 mm
ULCF life	8.7	5.7	1.8	1.7
*T*m	0.76	0.78	0.84	0.78

**Table 7:** Results of simulation of ULCF crack-initiation life for steel piers with corrosion pits in different positions and with different depth-to-diameter ratios.

Model	L1	L2	L3	P1	P2	P3
ULCF life	6.7	5.7	7.7	6.7	5.7	4.7
*T*m	0.76	0.69	0.79	0.66	0.69	0.67
Crack site	corner	corner	corner	pit	corner	corner

## Data Availability

The data presented in this study are available on request from the corresponding author. The data are not publicly available due to privacy.
